# Impact of *Piriformospora indica* on various characteristics of tomatoes during nickel nitrate stress under aeroponic and greenhouse conditions

**DOI:** 10.3389/fmicb.2022.1091036

**Published:** 2023-02-03

**Authors:** Nazanin Mahmoodi, Zahra Movahedi, Mehdi Ghabooli

**Affiliations:** Department of Plant Production and Genetics, Malayer University, Malayer, Iran

**Keywords:** nickel, *Piriformospora indica*, root, antioxidant enzymes, hydrogen peroxidase

## Abstract

As an essential nutrient for plant growth, nickel's (Ni) requirement is very low, and its augmented level causes environmental pollution and toxicity. Being a root endophytic fungus, *Piriformospora indica* (*P. indica*) can be beneficial to many plants under stress and non-stress conditions, particularly in terms of their improved growth performance. *P. indica*, as evidenced, enhances tolerance and resistance in most plants once they experience a range of stresses caused by biotic and abiotic factors, e.g., diseases and heavy metals. Against this background, the positive effects of *P. indica* on the tomato plants under Ni-induced stress (300, 600, and 900 mg L^−1^) were analyzed in three experiments at labs, at greenhouses, and *via* aeroponics in this study. The growth traits of the tomato plants, such as root length (RL) and root dry weight (RDW), were accordingly found to be positively boosted in the cases treated with *P. indica* compared to the non-treated ones. Treating with *P. indica* also thwarted the negative effects of Ni on some biochemical traits, including anthocyanin (Anth), proline (Pro), catalase (CAT), and glutathione peroxidase (GPx), while significantly minimizing the adverse impacts of this heavy metal at different levels on hydrogen peroxide (H_2_O_2_). Despite this, the Ni-stressed plants indicated much better traits in the presence of this fungus, compared with the non-treated ones, in most of the cases measured. Moreover, the photosynthetic pigments, i.e., chlorophyll a and b (Chl a & b) and carotenoid content (Carrot), were significantly higher in the tomato plants treated with *P. indica* under high Ni-induced stress as compared with the non-treated ones under non-Ni conditions, in which these pigments were low. The pro-production was further observed all through the *P. indica* inoculation, which could aid the treated plants in becoming Ni-stress-tolerant. Finally, the current study contributed to a better understanding of how to use the *P. indica* symbiosis to induce heavy metal tolerance in tomato plants, such as Ni, to meet the goals of sustainable agriculture.

## 1. Introduction

The association between the concentration of certain heavy metals in soils and plants has led to contamination that exceeds standard limits. The unprincipled disposal of sewage sludge and industrial effluents in farming fields has also given rise to nickel (Ni) accumulation in soils used for vegetable production and irrigated with untreated wastewater. Ni can thus harm some tissues, and it is further suspected of being responsible for some types of cancer. Moreover, no significant evidence indicates its nutritional value in higher organisms like humans (Ebrahimi et al., 2018). To date, no Ni-specific transporter has also been observed in plant cells for the uptake, but the Ni uptake has occurred by roots due to weak selective cation transporters, such as the Zrt/Irt-like proteins (ZIPs) (Van der Pas and Ingle, 2019). The increasing expression of various parts of the ZIP family has been additionally demonstrated in ribonucleic acid (RNA)-sequencing, whereas *Senecio coronatus* (Thunb.) Harv. in the family Asteraceae has shown the high expression of a ZIP transporter in roots, but this was not the considerable case in non-accumulating plants (Meier et al., 2018). However, the interpretation of the ZIP transporter behavior according to the sequence similarity models for plant ZIPs seems challenging due to the high rate of gene duplication in the ZIP family. The cytoplasmic chelation of Ni ions by ligands has also been introduced as one of the heavy-metal tolerance mechanisms, but the nature of the ligands, along with their importance, has not been identified. Besides, the amino acid histidine plays a significant role in the Ni transport from roots to shoots, owing to the high expression of the ATP-phosphoribosyl transferase in the amino acid histidine pathway (Sharma et al., 2021). Moreover, the epidermis of the shoot cells is the first location of Ni storage from the xylem, but the transporters for Ni unloading are not detected and may contain parts of the ZIP family (Visioli et al., 2014). Notably, the vacuole is the primary location of Ni deposition in cells.

Nevertheless, Ni has been spotted as an essential micronutrient for some microorganisms, such as algae, as it is used in some cofactors that are not detected in higher cells (Kumar et al., 2021). It is thus vital to plant growth and development and significantly contributes to various physiological processes, such as germination and yield performance. High Ni concentrations also change the metabolic processes of plants by preventing enzyme activity, inhibiting photosynthesis, and disturbing chlorophyll (Chl) biosynthesis (Shahzad et al., 2018). Under Ni-induced pollution, excess Ni, rather than a deficiency, is more commonly found. Its toxic effects on plants involve suppressed mitotic processes, lower plant growth rates, and reduced quantity and quality yield performance (Hassan et al., 2019).

Ni has been further mentioned to generate reactive oxygen species (ROS) in plants, thus causing oxidative stress and leading to the loss of biological processes as well as macromolecules (Barbouti et al., 2021). Even if plants have a defense mechanism to neutralize Ni-induced oxidative stress, such as antioxidant enzyme production, this defense system may not be adequate to moderate the impacts of Ni toxicity under high toxicity.

Notably, economic development has also contributed to environmental pollution by releasing high concentrations of toxic metals into the ecosystem, like Ni, which is a severe contaminant due to its widespread use in the agriculture and industry sectors (Rizwan et al., 2018). In Iran, good-quality tomatoes are grown in the open field because of a sunny environment and the differences between day and night temperatures. The application of phosphate fertilizers in tomato fields has thus led to Ni accumulation in the soils in this region and, consequently, the deposition of this heavy metal in tomatoes (Jalali and Karimi-Mojahed, 2020). The distribution of Ni in tomatoes is accordingly specified by the access to nutrients and the characteristics of soils and fertilizers applied for this purpose (Vittori-Antisari et al., 2015). Utilizing chemical fertilizers and disposing of industrial waste into the environment have further contaminated soils and water with Ni, which represents an environmental problem wherein this metal can be accumulated in crops and transferred to the food chain (Correia et al., 2018).

As evidenced, the inoculation of cherry tomato (*Lycopersicon esculentum*) with *Piriformospora indica* (*P. indica*), as a root endophytic fungus, had thus significantly improved tomato seedling growth under non-stress conditions (Varma et al., 2013; Anith et al., 2015). Nouh et al. (2020) additionally reported the positive effects of *P. indica* on salt stress tolerance and disease resistance in such plants. Similarly, Athira and Anith (2020) could increase the resistance of tomato plants to biotic stress caused by Fusarium wilt. Ghorbani et al. (2019) also observed the positive effects of *P. indica* on abiotic salinity tolerance and the growth of tomato plants under salinity conditions. Furthermore, *P. indica* has been so far used for heavy-metal toxicity tolerance in different plants, including tomatoes; for example, Baghaie and Aghili (2021) studied the effects of *P. indica* on lead toxicity in tomatoes and concluded that the antioxidant enzyme activities had been significantly amplified in roots in comparison to stems. However, it seems that the root treatment of different plants with useful fungi can promote some growth traits and enhance yield performance under non-stress conditions and even increase the resistance and tolerance of plants to biotic and abiotic stress, such as diseases and heavy metal toxicity (Aslam et al., 2019; Berni et al., 2019). Thus, this study investigated the effects of the root endophytic fungus, *P. indica*, on reducing Ni phytotoxicity in tomato plants, as the tomato fields are currently often being contaminated with sewage sludge and industrial effluents, and even irrigated with untreated wastewater.

## 2. Materials and methods

### 2.1. Fungus and plant inoculation

The fungus *P. indica* was gotten originally from the Department of Plant Production and Genetics, Faculty of Agriculture, Malayer University, Iran, and then grown on a complex medium. After the surface sterilization of the tomato cultivar ProSeed Urbana seeds with sodium hypochlorite (2%) for 10 min, they were germinated and inoculated with the suspension of *P. indica* spores and cultivated in pots containing sterile PittMoss. To generate spore suspension, the spores were released by gently scratching the fungus surface on the Petri dishes with a spatula. The spores were collected by centrifugation at 4000 rpm and then the pellet was rinsed three times in distilled water containing 0.02% Tween-20, sonicated to separate spores, and the spore concentration was finally adjusted to 5 × 105 spores per mL. Tomato seedlings were inoculated by immersing in the spore suspension solution with gentle shaking for 1–2 h in both experiments (soil and aeroponic culture). To prepare the fungal mycelium, the active disks were placed in a liquid medium and then incubated in a shaker incubator at 28 °C and 150 rpm for 7–10 days. Next, mycelium was filtered and washed several times with distilled water to remove the medium.

### 2.2. Experiments

#### 2.2.1. Lab germination

The tomato seeds were treated with 5.0% sodium hypochlorite for 5 min and then with 70.0% ethanol for 30 s for surface sterilization. Then, they were washed three times with distilled water. In total, 30 seeds of tomato were used for each Ni nitrate [Ni(NO_3_)_2_] stress treatment (0, 300, 600, and 900 mg L^−1^) and were placed in Petri dishes containing two layers of Whatman No 2.0 filter paper moistened with 10 mL of solutions or distilled water as the control. The stock solutions of Ni nitrate were further prepared in distilled water at the desired concentrations and diluted with distilled water to obtain suitable concentrations. Double-distilled water was applied as the control treatment. For inoculation, 2 g of mycelium were thoroughly mixed with 100 mL of extracted spore extract, and 2 mL were added to the Petri dishes, except for the control treatments without *P. indica*. The Petri dishes were then located in a growth cabinet with a temperature of 25°C and a 16/8-h light/dark cycle. The measured traits also included germination rate (GR), shoot fresh weight (SFW), fresh root weight (RFW), shoot dry weight (SDW), root dry weight (RDW), shoot length (SL), root length (RL), proline (Pro), flavonoid content (Flav), phenol content (Phen), catalase (CAT), and glutathione peroxidase (GPx).

#### 2.2.2. Greenhouse trial

Similar to the aeroponic system, the inoculated seeds with the fungus *P. indica* were transferred into the pots with PittMoss (Gramoflor GmbH & Co. KG, Germany) and soil after 14 days in the greenhouse. This soil mixture was also autoclaved two times with a one-day interval to sterilize. The nutrition was further performed with the 2 g L^−1^ Hoagland nutrient solution, and the Ni nitrate stress treatments (0, 300, 600, and 900 mg L^−1^) were applied after 3 weeks. The plants were grown in a growth chamber with 70% humidity, a temperature of 25°C, and a 16/8-h light/dark cycle under two different inoculating systems, including uninoculated and inoculated *P. indica*. Each treatment combination also consisted of four replication plants grown in separate pots. The seedlings were also harvested after 70 days, and the traits measured were RL, root volume (RV), stem length (SL), RDW, stem dry weight (SDW), dry leaf weight (LDW), and leaf number (LN). Besides, some biochemical traits were measured on the leaf samples, such as chlorophyll a (Chl a, b), carotenoid content (Carrot), Phen, protein content (Prot), anthocyanin (Anth), Pro, Flav, CAT, GPx, hydrogen peroxide (H_2_O_2_), and malondialdehyde (MDA).

#### 2.2.3. Aeroponic system

The aeroponic system was established in a growth chamber (each unit with 100 × 100 × 120 cm; depth × width × length) by controlling some environmental factors, such as temperature and photoperiod, which had a mobile front panel for managing and harvesting. The tomato roots were also sprayed with the Hoagland solution every 20 min for 20 s, the nutrient solution was renewed weekly, and the plants were staked out with a wire mesh if necessary. The *P. indica*-inoculated tomato seeds were further transferred into the aeroponic system after 14 days. The tomato seedlings were also maintained under environmental conditions with a 16/8 h photoperiod (day/night), 70% humidity, 350–400 μmol m^−2^ s^−1^ light intensity, and 25°C temperature. After 3 weeks, the *P. indica* infestation was verified, and the heavy-metal stress treatments (i.e., Ni nitrate) were applied in concentrations of 0, 300, 600, and 900 mg L^−1^. The seedlings were finally harvested after 40 days, and similar traits as in the greenhouse experiment were measured.

### 2.3. Protocols

All traits were measured in accordance with standard protocols. The seed germination properties were also recorded based on the International Rules for ISTA (1996). RL and SL were accordingly measured *via* a lab ruler, and RV was assessed with an overflow spout. The roots, stems, and leaves were also dried for 24 h separately, and then, RDW, SDW, and LDW were measured by a digital scale. In addition, LN per plant was counted in each experimental unit. The MDA content was further evaluated from the root tissue (Heath and Packer, 1968), and the Pro content was determined by Bates et al. (1973). Anth was additionally measured based on Gitelson et al. (2001), while the Flav estimation was done by the complementary colorimetric procedure introduced by Chang et al. (2002). The antioxidant enzymes were also assayed for CAT and GPx as well as for H_2_O_2_ with reference to the study of Mondal et al. (2004). Phen was further estimated based on the method developed by Zhu and Yao (2004), and total Prot was obtained according to the Bradford method (Kruger, 2009). The magnitudes of Chl a, b and Carrot were ultimately determined in line with Zhongfu et al. (2009).

### 2.4. Statistical analysis

The recorded datasets of three experiments were tested by the Anderson-Darling test for normality using the MINITAB (2017) (ver. 18.1) software package. The data from each experiment were also subjected to the analysis of variance (ANOVA) using Statistica version 10.0 (StatSoft, 2011). The SPSS version 23.0 (IBM, 2015) was also exercised to calculate the magnitude of the standard errors and the least significant difference (LSD) values (*p* < 0.05) if significant differences occurred in the treatment mean scores.

## 3. Results

### 3.1. Germination

**Figure 1:**
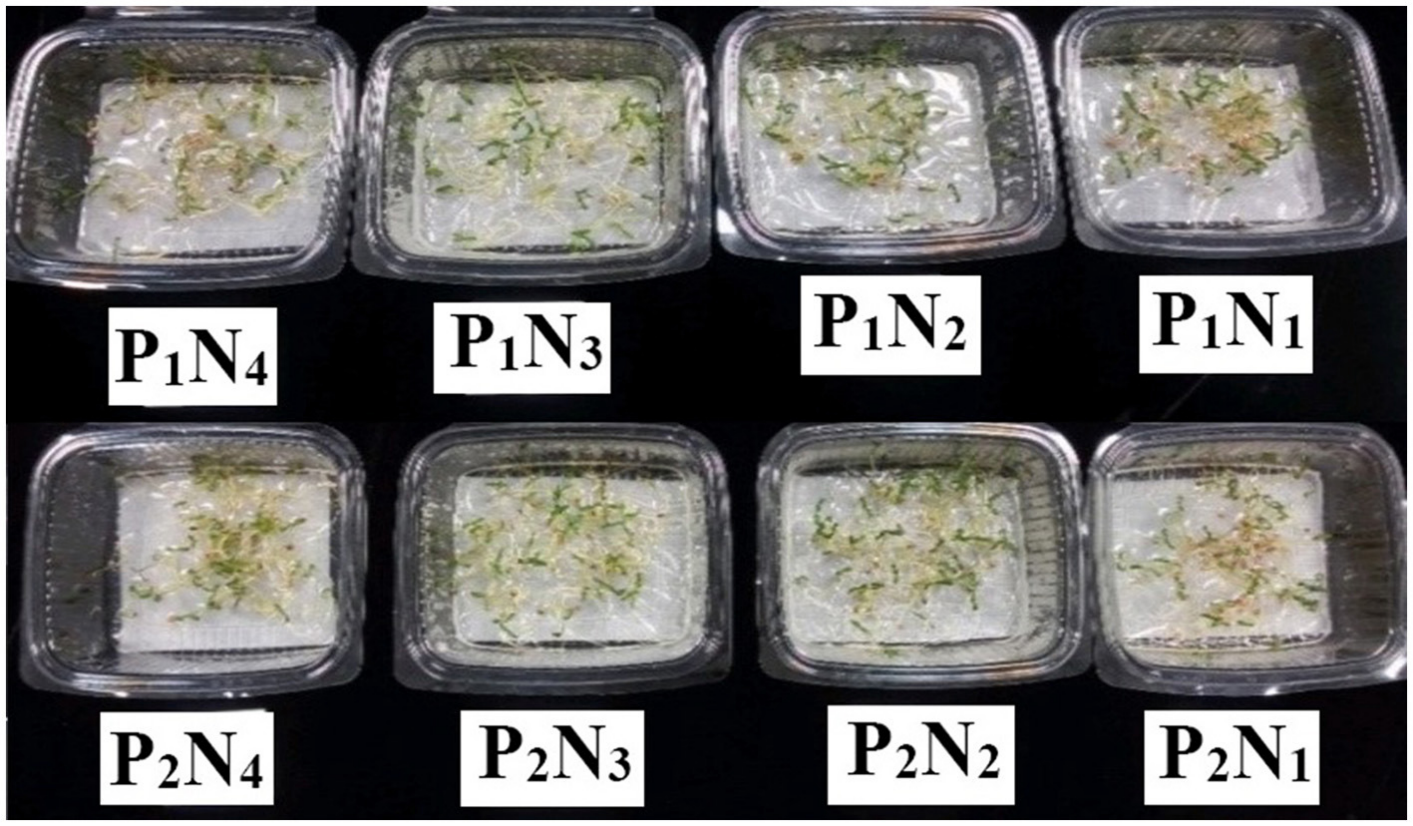
The germination experiment: the effect of *P. indica* treatment on the tomato germination under nickel stress, N_1_, N_2_, N_3_, and N_4_ (0, 300, 600, and 900 mg L^−1^). P_1_; untreated with *P. indica* and P_2_; treated with *P. indica*.

The ANOVA results showed a significant difference (*p* < 0.01) for the main effects of *P. indica* as well as for various concentrations of Ni in all measured traits (results are not shown). Moreover, the interaction effects of *P. indica* on Ni were significant (*p* < 0.01) for all measured traits except for SL, Phen, Pro, CAT, and GPx. The treatment with (results are not shown) 0.0 mg L^−1^ Ni nitrate and the non-treatment with *P. indica* were also assumed to be controls. Treating the tomato seeds with *P. indica* accordingly led to an increase of 32, 30, 69, 12, and 76% in RL, RFW, SFW, RDW, and SDW, respectively, over the non-treated control plants under non-Ni stress, while the amount of Flav decreased (21%) upon applying *P. indica* (Table 1). The treatment with *P. indica* also made Ni's harmful impacts ineffective in the other measured traits, including SL, SFW, GR, Pro, Phen, Flav, and GPx (Table 1). Thus, *P. indica* could stop the effects of 300 mg L^−1^ of Ni-induced stress on germination or even improve some traits (Figure 1).

**Table 1:** The effect of *P. indica* inoculation on the measured traits of tomato subjected to nickel nitrate stress under lab germination experiment.

	**RL (cm)**	**SL (cm)**	**RFW (g)**	**SFW (g)**	**RDW (g)**	**SDW (g)**
P_1_N_1_	6.50 ± 1.18^b^	3.11 ± 0.30^ab^	0.092 ± 0.001^b^	0.127 ± 0.074^b^	0.0082 ± 0.00013^b^	0.0520 ± 0.0008^b^
P_1_N_2_	6.24 ± 0.69^b^	2.82 ± 0.48^bc^	0.041 ± 0.008^e^	0.088 ± 0.013^bcde^	0.0022 ± 0.00013^d^	0.0062 ± 0.0017^d^
P_1_N_3_	4.34 ± 0.64^cd^	2.27 ± 0.14^d^	0.052 ± 0.008^d^	0.083 ± 0.006^ced^	0.0019 ± 0.00013^e^	0.0032 ± 0.0002^e^
P_1_N_4_	3.75 ± 0.38^d^	1.72 ± 0.14^e^	0.032 ± 0.001^f^	0.052 ± 0.014^e^	0.0013 ± 0.00008^f^	0.0012 ± 0.0013^f^
P_2_N_1_	8.59 ± 0.49^a^	3.41 ± 0.21^a^	0.120 ± 0.001^a^	0.215 ± 0.002^a^	0.0092 ± 0.00010^a^	0.0913 ± 0.0001^a^
P_2_N_2_	8.56 ± 0.23^a^	2.84 ± 0.16^bc^	0.062 ± 0.001^c^	0.120 ± 0.001^bc^	0.0027 ± 0.00013^c^	0.0415 ± 0.0001^c^
P_2_N_3_	5.17 ± 0.23^c^	2.69 ± 0.18^c^	0.062 ± 0.001^c^	0.092 ± 0.001^bcd^	0.0022 ± 0.00013^d^	0.0052 ± 0.0002^d^
P_2_N_4_	4.04 ± 0.19^d^	1.89 ± 0.26^ed^	0.052 ± 0.001^d^	0.072 ± 0.001^de^	0.0013 ± 0.00010^f^	0.0031 ± 0.0001^e^
	**GPX** μ**mol g**^−1^ **FW**^−1^ **min**^−1^	**CAT** μ**mol g**^−1^ **FW**^−1^ **min**^−1^	**GR** **seed hour**^−1^	**Pro** μ**mol g**^−1^ **FW**^−1^	**Flav** μ**mol g**^−1^ **FW**^−1^	**Phen** **mg g**^−1^ **DW**^−1^
P_1_N_1_	4.83 ± 0.10^ab^	6.02 ± 0.87^a^	2.22 ± 0.12^ab^	0.1331 ± 0.0087^e^	0.0153 ± 0.0004^c^	0.1086 ± 0.0056^d^
P_1_N_2_	4.97 ± 0.05^ab^	4.25 ± 0.47^b^	2.12 ± 0.08^b^	0.2051 ± 0.0187^bc^	0.0159 ± 0.0013^c^	0.1298 ± 0.0058^bc^
P_1_N_3_	5.83 ± 0.02^a^	4.09 ± 0.30^bc^	1.73 ± 0.13^c^	0.2393 ± 0.0200^ab^	0.0190 ± 0.0004^b^	0.1470 ± 0.0093^a^
P_1_N_4_	5.30 ± 0.08^ab^	3.30 ± 0.47^c^	1.54 ± 0.11^c^	0.2819 ± 0.0143^a^	0.0206 ± 0.0016^a^	0.1565 ± 0.0083^a^
P_2_N_1_	4.35 ± 0.04^b^	5.85 ± 0.52^a^	2.32 ± 0.15^a^	0.1364 ± 0.0262^de^	0.0126 ± 0.0003^d^	0.1012 ± 0.0055^d^
P_2_N_2_	4.66 ± 2.65^ab^	5.31 ± 0.54^a^	2.22 ± 0.17^ab^	0.1786 ± 0.0230^cd^	0.0166 ± 0.0008^c^	0.1170 ± 0.0149^cd^
P_2_N_3_	3.95 ± 0.49^b^	4.25 ± 0.65^b^	2.10 ± 0.21^b^	0.2352 ± 0.0364^b^	0.0184 ± 0.0009^b^	0.1255 ± 0.0072^c^
P_2_N_4_	5.05 ± 0.33^ab^	3.70 ± 0.31^bc^	1.72 ± 0.06^c^	0.2792 ± 0.0586^a^	0.0193 ± 0.0015^ab^	0.1434 ± 0.0222^ab^

Means (±SD, *n* = 4) with the same letter are not different significantly according to LSD test (*P* < 0.05).

Treatments are: P_1_, untreated with *P. indica* and P_2_, treated with *P. indica*; N_1_, 0 mg L^−1^ nickel stress; N_2_, 300 mg L^−1^ nickel stress; N_3_, 600 mg L^−1^ nickel stress; and N_4_, 900 mg L^−1^ nickel stress.

SL, shoot length; RL, root length; RFW, root fresh weight; SFW, shoot fresh weight; RDW, root dry weight; SDW, shoot dry weight; CAT, catalase; GPX, glutathione peroxidase; GR, germination rate; Pro, proline; Flav, flavonoid content; Phen, phenol content.

Under 600 mg L^−1^ of Ni, several germination traits such as SL, RFW, EDW, SDW, and GR were elevated due to the application of *P. indica* (Table 1). When subjected to severe stress from Ni (900 mg L^−1^), RFW and SDW also elevated in the *P. indica*-treated group, while such a treatment deteriorated the adverse effects of Ni-induced stress on the other recorded traits at the germination stage (Table 1). Using *P. indica* enhanced tomato seed germination under non-stress conditions. It thwarted its harmful effects under different levels of Ni stress on various seed germination properties.

### 3.2. Greenhouse seedling

**Figure 2:**
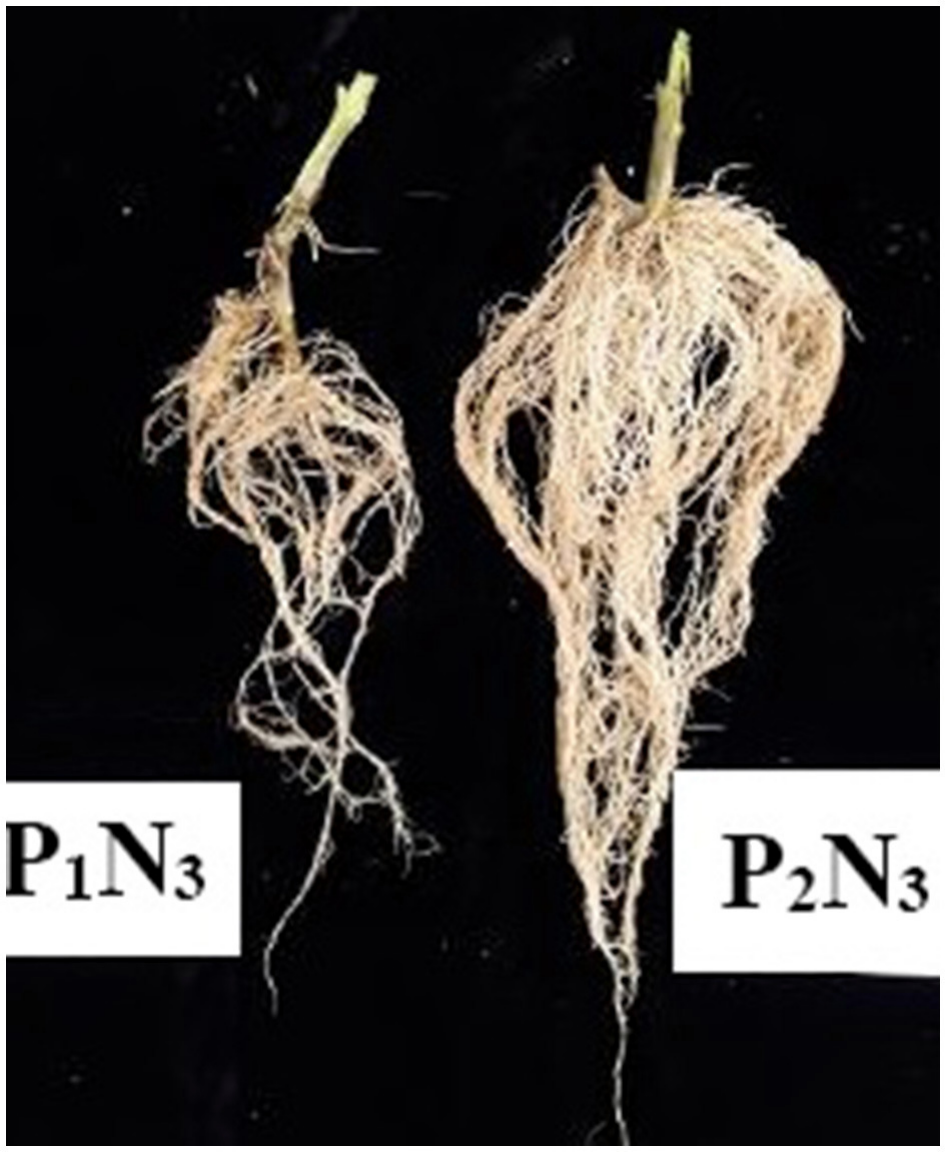
The greenhouse experiment: the effect of *P. indica* treatment on the tomato root under nickel stress, N_3_ (600 mg L^−1^). P_1_; untreated with *P. indica* and P_2_; treated with *P. indica*.

The ANOVA results indicated significant differences (*p* < 0.01) for the main effects of *P. indica* and Ni on all measured traits, whereas their interaction effects were only significant (*p* < 0.01) for SL, RV, RDW, Chl b, and GPx. The treatment with 0.0 mg L^−1^ Ni nitrate and the non-treatment with *P. indica* were also assumed as the controls. In the greenhouse experiment, the *P. indica*-treated plants showed a significant increase in RL (20%), RV (56%), RDW (46%), SDW (33%), LDW (30%), LN (22%), CAT (21%), GPx (29%), Prot (15%), and Anth (4%) under non-stress conditions, while the magnitudes of Phen, Pro, and Chl a & b did not alter following the *P. indica* application (Table 2). Under 300 mg L^−1^ Ni stress, the magnitudes of RV (35%), SDW (21%), LDW (29%), Prot (69%), Anth (15%), Carrot (108%), and Chl b (105%) also enlarged due to the *P. indica* use (Table 2). Moreover, applying *P. indica* caused the negative impacts of Ni to be ineffective in RL, RDW, LN, CAT, GPx, and Chl a (Table 2). Under 600 mg L^−1^ of Ni stress, the quantities of RL (36%), RV (20%), RDW (44%), SDW (32%), LDW (33%), Prot (91%), Anth (5%), and Carrot (39%) correspondingly enhanced due to the *P. indica* application (Figure 2).

**Table 2:** The effect of *P. indica* inoculation on the measured traits of tomato subjected to nickel nitrate stress under greenhouse experiment.

	**RL cm**	**RV cm^−3^**	**RDW g**	**SDW g**	**LDW g**	**LN No**.
P_1_N_1_	43.4 ± 8.0^b^	29.3 ± 4.3^b^	4.1 ± 0.66^b^	13.8 ± 1.12^b^	12.3 ± 0.57^b^	220.5 ± 24.1^b^
P_1_N_2_	45.3 ± 2.8^b^	17.1 ± 5.4^d^	2.8 ± 0.38^c^	9.8 ± 0.60^d^	8.2 ± 0.66^d^	153.8 ± 28.8^dc^
P_1_N_3_	32.3 ± 1.8^dc^	12.0 ± 2.5^ef^	1.8 ± 0.29f^e^	7.7 ± 1.79^e^	6.7 ± 0.27^e^	132.8 ± 14.5^def^
P_1_N_4_	29.0 ± 3.7^d^	9.6 ± 2.6^f^	1.3 ± 0.43^f^	7.2 ± 0.99^e^	5.8 ± 0.33^e^	117.0 ± 8.8^f^
P_2_N_1_	51.9 ± 2.6^a^	45.6 ± 2.2^a^	6.0 ± 0.43^a^	18.3 ± 0.59^a^	16.0 ± 0.19^a^	269.0 ± 14.4^a^
P_2_N_2_	45.7 ± 2.9^b^	23.1 ± 1.1^c^	3.0 ± 0.26^c^	11.9 ± 0.87^c^	10.6 ± 0.33^c^	172.8 ± 8.7^c^
P_2_N_3_	43.8 ± 1.1^b^	14.4 ± 1.1^de^	2.6 ± 0.29^cd^	10.2 ± 0.66^d^	8.9 ± 0.33^d^	150.3 ± 10.4^cde^
P_2_N_4_	36.2 ± 4.6^c^	14.5 ± 1.4^de^	2.0 ± 0.43^de^	7.4 ± 0.61^e^	6.8 ± 0.33^e^	129.5 ± 8.4^fe^
	**H**_2_**O**_2_ **nmol g**^−1^ **FW**^−1^	**Phen** **mg g**^−1^ **DW**^−1^	**Flav** μ**mol g**^−1^ **FW**^−1^	**MDA** μ**mol g**^−1^ **FW**^−1^	**CAT** μ**mol g**^−1^ **FW**^−1^ **min**^−1^	**GPX** μ**mol g**^−1^ **FW**^−1^ **min**^−1^
P_1_N_1_	0.1069 ± 0.00023^e^	0.069 ± 0.0008^c^	0.0106 ± 0.00068^c^	2.33 ± 0.338^c^	2.85 ± 0.15^c^	2.68 ± 0.84^c^
P_1_N_2_	0.1136 ± 0.00030^d^	0.072 ± 0.0003^b^	0.0114 ± 0.00030^c^	2.78 ± 0.270^b^	3.75 ± 0.21^b^	3.85 ± 0.82^b^
P_1_N_3_	0.1155 ± 0.00024^c^	0.075 ± 0.0016^a^	0.0166 ± 0.00074^a^	3.27 ± 0.150^a^	3.66 ± 0.26^b^	3.73 ± 1.47^b^
P_1_N_4_	0.1234 ± 0.00064^a^	0.074 ± 0.0007^a^	0.0177 ± 0.00025^a^	3.20 ± 0.113^a^	4.26 ± 0.14^a^	4.50 ± 0.94^a^
P_2_N_1_	0.1040 ± 0.00059^f^	0.068 ± 0.0007^c^	0.0083 ± 0.00032^d^	1.52 ± 0.085^d^	3.46 ± 0.20^b^	3.47 ± 0.60^b^
P_2_N_2_	0.1080 ± 0.00024^e^	0.069 ± 0.0005^c^	0.0092 ± 0.00130^d^	2.13 ± 0.253^c^	3.44 ± 0.23^b^	3.44 ± 1.25^b^
P_2_N_3_	0.1126 ± 0.00024^d^	0.074 ± 0.0019^a^	0.0131 ± 0.00147^b^	2.31 ± 0.164^c^	3.79 ± 0.18^b^	3.89 ± 0.92^b^
P_2_N_4_	0.1177 ± 0.00036^b^	0.072 ± 0.0015^b^	0.0137 ± 0.00060^b^	2.40 ± 0.193^c^	3.89 ± 0.14^b^	4.03 ± 0.56^a^
	**Prot** **mg g**^−1^ **FW**^−1^	**Pro** μ**mol g**^−1^ **FW**^−1^	**Ant** **Abs 530 g**^−1^ **FW**^−1^	**Carot** **mg g**^−1^ **FW**^−1^	**Chl a** **mg g**^−1^ **FW**^−1^	**Chl b** **mg g**^−1^ **FW**^−1^
P_1_N_1_	0.0089 ± 0.0008^b^	0.060 ± 0.020^cd^	180.9 ± 2.12^b^	11.2 ± 1.07^ab^	1.46 ± 0.33^a^	4.13 ± 0.03^a^
P_1_N_2_	0.0076 ± 0.0007^cd^	0.078 ± 0.011^bc^	159.9 ± 0.55^d^	9.7 ± 0.52^c^	1.04 ± 0.06^b^	2.57 ± 0.29^b^
P_1_N_3_	0.0049 ± 0.0011^f^	0.140 ± 0.014^a^	158.7 ± 1.12^de^	5.1 ± 0.66^e^	0.79 ± 0.11^cb^	2.11 ± 0.56^cb^
P_1_N_4_	0.0045 ± 0.0006^f^	0.109 ± 0.016^ab^	135.4 ± 2.10^f^	4.9 ± 0.76^e^	0.40 ± 0.31^d^	1.85 ± 0.49^c^
P_2_N_1_	0.0102 ± 0.0008^a^	0.041 ± 0.028^cd^	188.8 ± 0.82^a^	12.2 ± 0.48^a^	1.74 ± 0.30^a^	4.45 ± 0.04^a^
P_2_N_2_	0.0083 ± 0.0008^cb^	0.034 ± 0.058^d^	183.2 ± 0.24^b^	10.6 ± 0.69^bc^	1.11 ± 0.05^b^	4.32 ± 0.30^a^
P_2_N_3_	0.0068 ± 0.0006^de^	0.074 ± 0.021^bcd^	166.8 ± 2.86^c^	7.1 ± 0.41^d^	1.06 ± 0.28^b^	2.44 ± 0.34^b^
P_2_N_4_	0.0062 ± 0.0006^e^	0.058 ± 0.033^cd^	154.1 ± 1.67^e^	5.5 ± 0.62^e^	0.71 ± 0.11^cd^	2.30 ± 0.19^cb^

Means (±SD, *n* = 4) with the same letter are not different significantly according to LSD test (*P* < 0.05). Treatments are: P_1_, untreated with *P. indica* and P_2_, treated with *P. indica*; N_1_, 0 mg L^−1^ nickel stress; N_2_, 300 mg L^−1^ nickel stress; N_3_, 600 mg L^−1^ nickel stress; and N_4_, 900 mg L^−1^ nickel stress.

RL, root length; RV, root volume; RDW, root dry weight; SDW, stem dry weight; LDW, leaf dry weight; LN, leaf number; H_2_O_2_, hydrogen peroxide; Phen, phenol content; Flav, flavonoid content; MDA, malondialdehyde; CAT, catalase; GPX, glutathione peroxidase; Prot, protein content; Pro, proline; Anth, anthocyanin; Carot, carotenoid content; Chl a, chlorophyll a; Chl b, chlorophyll b.

Under severe stress from Ni (900 mg L^−1^), the positive effects of *P. indica* were restricted to RL (25%), RV (51%), RDW (54%), and Anth (14%), while the use of *P. indica* frustrated the harmful impacts of Ni-induced stress on SDW, LDW, LN, GPx, Carrot, and Chl a, b (Table 2). In contrast, using *P. indica* could not eliminate the harmful effects of Ni stress at high concentrations (900 mg L^−1^) in H_2_O_2_, Phen, Flav, MDA, CAT, Prot, and Pro (Table 2). The activity of the GPx antioxidant enzyme did not also decrease, especially under severe Ni stress, denoting that certain rootstocks could mitigate Ni toxicity *via* detoxification in cells as a process toward the detoxification of H_2_O_2_ and lipid peroxides by reduced glutathione.

### 3.3. Aeroponic system

The ANOVA results demonstrated significant differences (*p* < 0.01) for the main effects of *P. indica* and Ni in all measured traits. Besides, the interaction effects of *P. indica* by Ni were significant (*p* < 0.01) for all measured traits except for Anth. The treatment with 0.0 mg L^−1^ Ni nitrate and the non-treatment with *P. indica* were also assumed to be the controls. In the aeroponic experiment, the *P. indica*-treated plants accordingly indicated a significant upsurge in RL (17%), RV (12%), RDW (49%), SDW (35%), LDW (28%), LN (37%), CAT (22%), GPx (25%), Prot (25%), and Anth (10%) under non-stress conditions, while the magnitudes of MDA, Carrot, and Chl a & b did not change after treatment with *P. indica* (Table 3). Under 300 mg L^−1^ Ni-induced stress, the amounts of RL (19%), RV (7%), RDW (72%), SDW (25%), LDW (14%), LN (70%), Prot (27%), and Anth (13%) also elevated due to the *P. indica* application (Table 3).

**Table 3:** The effect of *P. indica* inoculation on the measured traits of tomato subjected to nickel nitrate stress under aeroponic experiment.

	**RL cm**	**RV cm^−3^**	**RDW g**	**SDW g**	**LDW g**	**LN No**.
P_1_N_1_	81.0 ± 0.95^b^	199.7 ± 1.42^c^	14.6 ± 0.48^b^	15.7 ± 0.34^b^	14.5 ± 0.17^b^	46.5 ± 2.7^c^
P_1_N_2_	65.4 ± 0.93^d^	192.7 ± 1.71^d^	12.7 ± 0.95^c^	11.6 ± 0.84^d^	10.5 ± 0.32^d^	31.5 ± 1.3^ed^
P_1_N_3_	59.0 ± 0.57^f^	134.1 ± 2.47^h^	7.7 ± 0.39^e^	9.5 ± 0.24^e^	8.7 ± 0.17^e^	30.5 ± 2.5^e^
P_1_N_4_	55.6 ± 0.50^g^	180.8 ± 0.90^f^	8.4 ± 0.75^e^	6.4 ± 0.17^g^	5.2 ± 0.82^g^	19.5 ± 1.8^f^
P_2_N_1_	94.5 ± 0.96^a^	222.8 ± 1.94^a^	21.7 ± 0.17^a^	21.2 ± 0.21^a^	18.6 ± 0.26^a^	63.8 ± 1.3^a^
P_2_N_2_	77.8 ± 1.09^c^	207.1 ± 1.11^b^	21.8 ± 0.42^a^	14.5 ± 0.40^c^	12.0 ± 0.42^c^	53.5 ± 2.1^b^
P_2_N_3_	65.1 ± 1.21^d^	156.6 ± 0.93^g^	10.8 ± 0.13^d^	11.5 ± 0.13^d^	10.6 ± 0.73^d^	33.3 ± 1.7^d^
P_2_N_4_	63.0 ± 1.24^e^	185.9 ± 1.06^e^	12.2 ± 0.26^c^	7.3 ± 0.18^f^	8.0 ± 0.22^f^	19.3 ± 1.3^f^
	**H**_2_**O**_2_ **nmol g**^−1^ **FW**^−1^	**Phenol** **mg g**^−1^ **DW**^−1^	**Flav** μ**mol g**^−1^ **FW**^−1^	**MDA** μ**mol** **g**^−1^ **FW**^−1^	**CAT** μ**mol g**^−1^ **FW**^−1^ **min**^−1^	**GPX** μ**mol g**^−1^ **FW**^−1^ **min**^−1^
P_1_N_1_	0.1065 ± 0.0003^e^	0.0975 ± 0.0014^e^	0.0135 ± 0.0012^c^	1.95 ± 0.18^d^	2.69 ± 0.21^c^	2.57 ± 0.31^c^
P_1_N_2_	0.1122 ± 0.0001^c^	0.1075 ± 0.0022^d^	0.0169 ± 0.0004^b^	2.79 ± 0.09^b^	3.94 ± 0.61^b^	3.46 ± 0.48^b^
P_1_N_3_	0.1177 ± 0.0003^b^	0.0698 ± 0.0021^g^	0.0198 ± 0.0004^a^	3.22 ± 0.13^a^	4.12 ± 0.79^b^	3.61 ± 0.07^b^
P_1_N_4_	0.1234 ± 0.0003^a^	0.1662 ± 0.0015^a^	0.0210 ± 0.0018^a^	2.71 ± 0.17^b^	4.84 ± 0.31^a^	4.94 ± 0.03^a^
P_2_N_1_	0.1046 ± 0.0004^f^	0.0928 ± 0.0018^f^	0.0109 ± 0.0009^d^	1.75 ± 0.28^d^	3.27 ± 0.47^b^	3.22 ± 0.72^b^
P_2_N_2_	0.1063 ± 0.0020^e^	0.1041 ± 0.0032^d^	0.0142 ± 0.0001^c^	2.33 ± 0.18^c^	3.15 ± 0.57^b^	3.73 ± 0.29^b^
P_2_N_3_	0.1109 ± 0.0011^d^	0.1170 ± 0.0054^c^	0.0161 ± 0.0004^b^	3.18 ± 0.46^a^	3.41 ± 0.36^b^	3.81 ± 0.31^b^
P_2_N_4_	0.1181 ± 0.0007^b^	0.1280 ± 0.0047^b^	0.0200 ± 0.0005^a^	3.01 ± 0.14^ab^	3.62 ± 0.52^b^	4.17 ± 0.16^a^
	**Protein** **mg g**^−1^ **FW**^−1^	**Proline** μ**mol g**^−1^ **FW**^−1^	**Ant** **Abs 530 g**^−1^ **FW**^−1^	**Carot** **mg g**^−1^ **FW**^−1^	**Chl a** **mg g**^−1^ **FW**^−1^	**Chl b** **mg g**^−1^ **FW**^−1^
P_1_N_1_	0.0112 ± 0.0007^b^	0.106 ± 0.009^de^	178.7 ± 2.07^b^	12.56 ± 1.04^a^	2.27 ± 0.24^a^	4.22 ± 0.49^a^
P_1_N_2_	0.0074 ± 0.0005^d^	0.124 ± 0.010^cd^	162.1 ± 0.65^c^	9.59 ± 2.41^b^	2.32 ± 0.11^a^	3.05 ± 0.15^b^
P_1_N_3_	0.0046 ± 0.0015^f^	0.147 ± 0.009^bc^	153.6 ± 3.77^d^	7.07 ± 0.35^dc^	1.13 ± 0.06^b^	0.85 ± 0.41^d^
P_1_N_4_	0.0033 ± 0.0005^g^	0.291 ± 0.019^a^	136.6 ± 9.74^e^	5.76 ± 0.62^d^	1.32 ± 0.04^b^	0.71 ± 0.23^d^
P_2_N_1_	0.0140 ± 0.0006^a^	0.023 ± 0.014^f^	196.8 ± 0.77^a^	13.84 ± 0.26^a^	2.21 ± 0.05^a^	3.77 ± 0.40^a^
P_2_N_2_	0.0094 ± 0.0007^c^	0.068 ± 0.014^e^	183.0 ± 1.29^b^	10.96 ± 0.80^b^	2.16 ± 0.25^a^	3.23 ± 0.06^b^
P_2_N_3_	0.0088 ± 0.0001^c^	0.122 ± 0.044^cd^	156.4 ± 1.96^cd^	9.92 ± 0.36^b^	2.17 ± 0.09^a^	1.55 ± 0.38^c^
P_2_N_4_	0.0060 ± 0.0008^e^	0.170 ± 0.053^b^	160.6 ± 8.97^cd^	7.79 ± 0.36^c^	1.33 ± 0.11^b^	1.76 ± 0.32^c^

Means (±SD, *n* = 4) with the same letter are not different significantly according to LSD test (*P* < 0.05).

Treatments are: P_1_, untreated with *P. indica* and P_2_, treated with *P. indica*; N_1_, 0 mg L^−1^ nickel stress; N_2_, 300 mg L^−1^ nickel stress; N_3_, 600 mg L^−1^ nickel stress; and N_4_, 900 mg L^−1^ nickel stress.

RL, root length; RV, root volume; RDW, root dry weight; SDW, stem dry weight; LDW, leaf dry weight; LN, leaf number; H_2_O_2_, hydrogen peroxide; Phen, phenol content; Flav, flavonoid content; MDA, malondialdehyde; CAT, catalase; GPX, glutathione peroxidase; Prot, protein content; Pro, proline; Anth, anthocyanin; Carot, carotenoid content; Chl a, chlorophyll a; Chl b, chlorophyll b.

**Figure 3:**
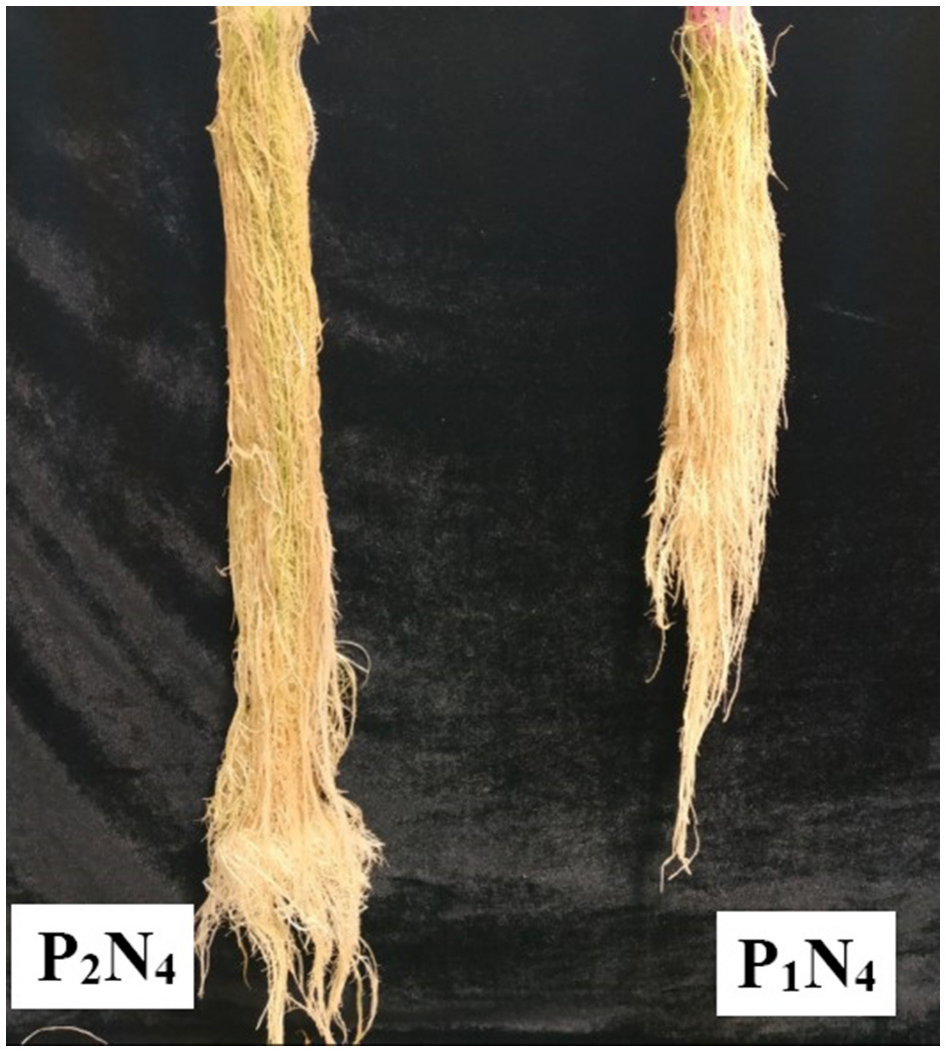
The aeroponic experiment: the effect of *P. indica* treatment on the tomato root under nickel stress, N_4_ (900 mg L^−1^). P_1_; untreated with *P. indica* and P_2_; treated with *P. indica*.

Under the 600 mg L^−1^ of Ni stress, RL (10%), RV (17%), RDW (40%), SDW (21%), LFW (22%), LN (9%), Phen (68%), Prot (91%), Carrot (40%), Chl a (92%), and Chl b (82%) also augmented as the result of using *P. indica* (Table 3). Under severe stress from Ni (900 mg L^−1^), the positive effects of *P. indica* were also limited to RL (13%), RV (3%), RDW (45%), SDW (14%), LDW (54%), Prot (82%), and Chl b (148%), while the application of *P. indica* blocked the adverse impacts of Ni stress on LN, Flav, MDA, GPx, and Chl a (Figure 3).

### 3.4. Trait associations

**Figure 4:**
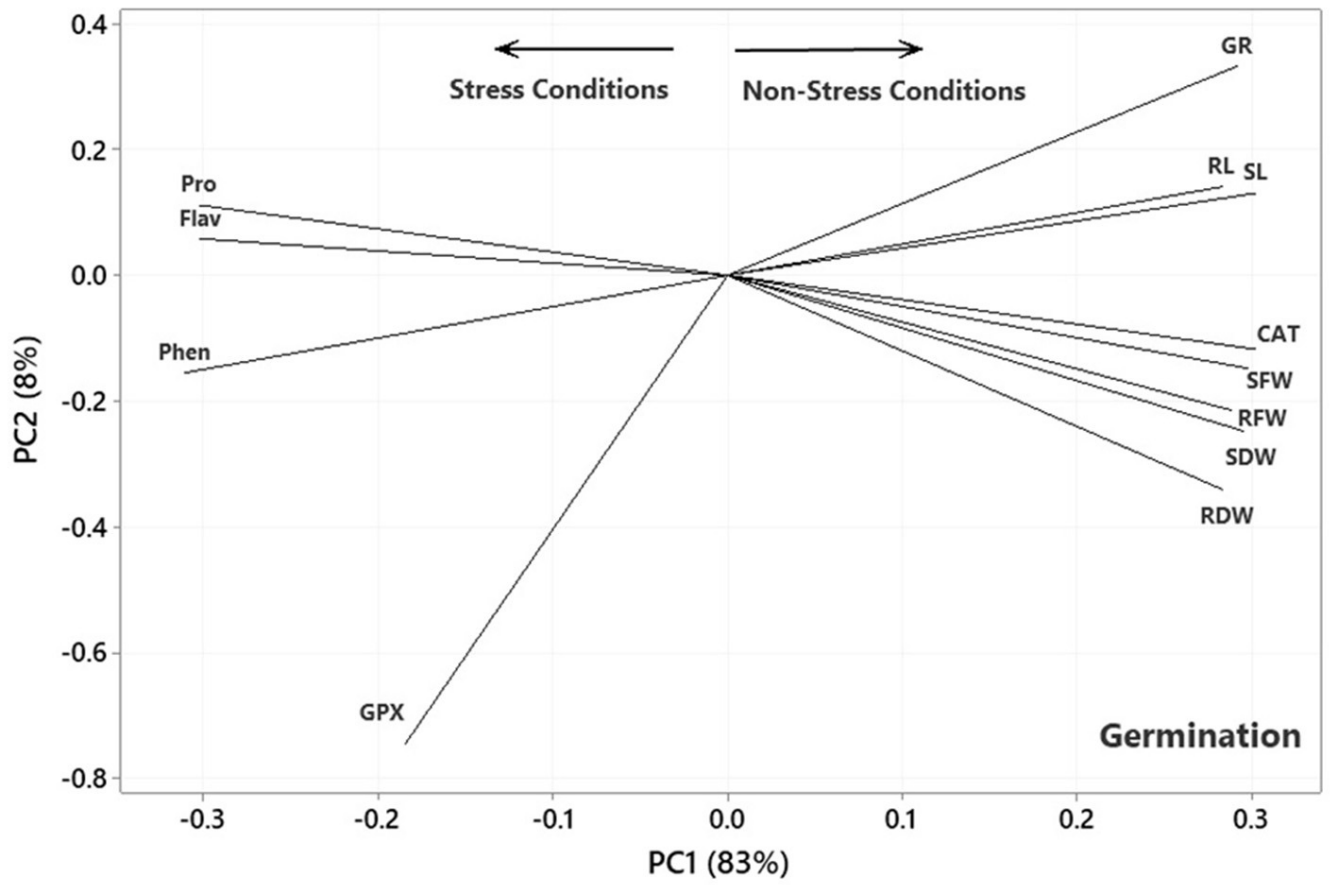
Plot showing the association among the measured traits subjected to four treatments (0, 300, 600, and 900 mg L^−1^) in the germination experiment based on the two first PCs. SL, shoot length; RL, root length; RFW, root fresh weight; SFW, shoot fresh weight; RDW, root dry weight; SDW, shoot dry weight; CAT, catalase; GPX, glutathione peroxidase; GR, germination rate; Pro, proline; Flav, flavonoid content; Phen, phenol content.

The results of the principal component analysis (PCA) based on the correlation matrices indicated that the two first PCs described 91, 92, and 90% of the variance in the lab germination, greenhouse, and aeroponic experiments, respectively. In the plot of the germination (Figure 4), the PC1 axis mainly distinguished the traits of Pro, Flav, Phen, and GPx from the others. Thus, the first PC could separate the traits into two main groups according to the Ni-induced stress conditions. Figure 4 shows the traits corresponding to Ni stress on the left and those based on normal or non-stress conditions, except for CAT, on the right. It further facilitates the visualization of the associations among the traits by approximating the correlation coefficient through the cosine of the angle between the vectors. Therefore, some of the most prominent associations, according to Figure 4, were as follows: (i) a strong positive association between RL and SL and between Pro and Flav, as illustrated by the small acute angles between their vectors (*r* = cos 0 = +1), (ii) a near-zero correlation between GPx and Pro and Flav, as indicated by the near perpendicular vectors (*r* = cos 90 = 0), and (iii) a strong negative association between Phen and RL and SL, and between CAT and SFW and Pro and Flav, as indicated by the large obtuse angles (*r* = cos 180 = −1).

**Figure 5:**
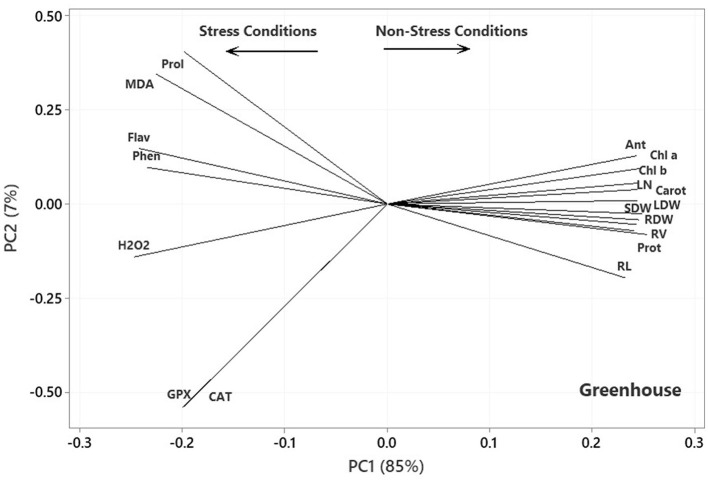
Plot showing the association among the measured traits subjected to four treatments (0, 300, 600, and 900 mg L^−1^) in the greenhouse experiment based on the two first PCs. RL, root length; RV, root volume; RDW, root dry weight; SDW, stem dry weight; LDW, leaf dry weight; LN, leaf number; H_2_O_2_, hydrogen peroxide; Phen, phenol content; Flav, flavonoid content; MDA, malondialdehyde; CAT, catalase; GPX, glutathione peroxidase; Prot, protein content; Pro, proline; Anth, anthocyanin; Carot, carotenoid content; Chl a, chlorophyll a; Chl b, chlorophyll b.

**Figure 6:**
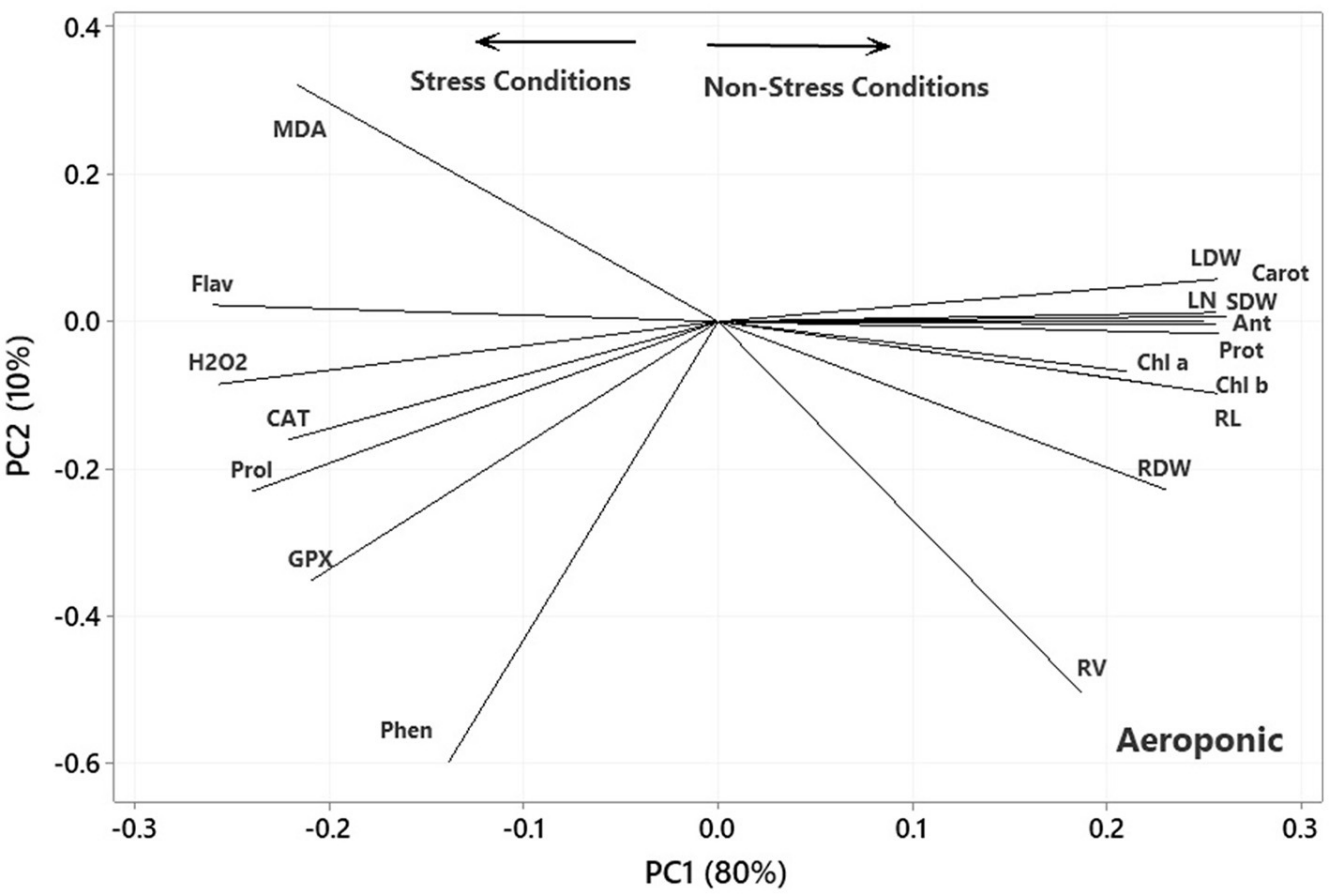
Plot showing the association among the measured traits subjected to four treatments (0, 300, 600, and 900 mg L^−1^) in the aeroponic experiment based on the two first PCs. RL, root length; RV, root volume; RDW, root dry weight; SDW, stem dry weight; LDW, leaf dry weight; LN, leaf number; H_2_O_2_, hydrogen peroxide; Phen, phenol content; Flav, flavonoid content; MDA, malondialdehyde; CAT, catalase; GPX, glutathione peroxidase; Prot, protein content; Pro, proline; Anth, anthocyanin; Carot, carotenoid content; Chl a, chlorophyll a; Chl b, chlorophyll b.

In the plot of the greenhouse experiment (Figure 5), the PC1 axis could further split the traits of Pro, MDA, Flav, Phen, H_2_O_2_, CAT, and GPx from the others. Thus, the PC1 could separate the traits based on Ni-induced stress, like that in the germination, even better due to the standing of CAT near other traits related to the stress conditions. Moreover, some of the most prominent relationships based on Figure 5 were (i) a strong positive association between CAT and GPx and between Phen and Flav, (ii) a near-zero correlation between GPx and CAT and Pro and MDA, and (iii) a strong negative association between RL and Phen and Flav, between H_2_O_2_ and Anth and Chl a & b. Most of the mentioned relationship predictions could also be verified from the original data because the PCA described the bulk of the total variances (over 90%). Figure 6 also shows that the PC1 axis sets apart the same traits in both aeroponic and greenhouse experiments, i.e., Pro, MDA, Flav, Phen, H_2_O_2_, CAT, and GPx in one group and the other traits in another group. Therefore, the PC1 could discriminate the traits based on the stress conditions in the aeroponic experiment, like those in lab germination and greenhouse ones. Some of the most prominent correlations based on Figure 6 were as follows: (i) a strong positive correlation between LN, Anth, and Carrot and Prot, and between CAT and Pro, (ii) a near-zero correlation between Phen and MDA, and (iii) a strong negative association between RV and MDA, and between LDW and SDW and Flav.

## 4. Discussion

The study results established that increasing Ni-induced stress from 0 to 900 mg L^−1^ reduced most traits in tomato plants due to the phytotoxicity of Ni stress. Similar results have been further reported in this respect (Nazir et al., 2019; Jahan et al., 2020). Notably, the Ni requirement in plants is very low, and its toxicity happens at 10 mg g^−1^ for the sensitive species, but the tolerant ones can bear 50 mg g^−1^ concentrations (Kumar et al., 2021). Hassan et al. (2019) had accordingly shown that the negative effects of Ni on plants could be due to the reduced uptake and the translocation of essential nutrients to their shoots induced by Ni phytotoxicity. The effects of Ni on tomato growth could be further related to the enhancement of lipid peroxidation because heavy metal ions can initiate lipid peroxidation in plants (Giannakoula et al., 2021). As the MDA production, the magnitude of lipid peroxidation was also elevated in the higher levels of Ni stress compared to the lower levels or under non-stress conditions.

**Figure 7:**
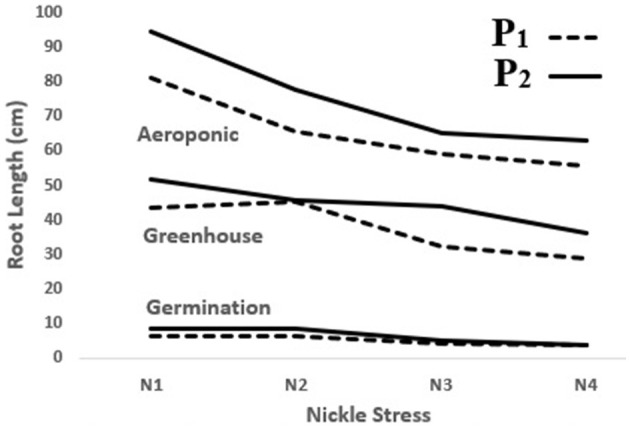
The effect of *P. indica* treatment on root length in the tomato subjected to nickel exposure (0, 300, 600, and 900 mg L^−1^). P_1_; untreated with *P. indica* and P_2_; treated with *P. indica*.

In the present study, the significant, positive effect of the *P. indica* application on decelerating depression in most of the measured traits was observed in the lab, greenhouse, and aeroponic experiments, whereas RL was enhanced by 32, 20, and 17% following the *P. indica* use in comparison with non-Ni stress in the germination, greenhouse, and aeroponic experiments, respectively. In other words, *P. indica* application could decrease the rate of trait depression under Ni stress (Figure 7). Similarly, Bagde et al. (2011) reported that treating sunflowers with *P. indica* increased RL by 36% and RDW by 24%. Usman et al. (2019) further stated that the root and shoot characteristics had been harmfully affected by Ni stress because of Ni toxicity disorders in the metabolic system and had thus prevented the normal cell cycle process.

This study revealed that the tomato plants could withstand up to 300 or even 600 mg L^−1^ of Ni stress after the *P. indica* treatment and exhibited higher growth traits than the non-treated ones, such as RL and SL. In other words, applying *P. indica* could maintain the tomato plant's tolerance to Ni-induced stress in morphological traits and remove the harmful effects of heavy metal contamination. According to Sepehri and Khatabi (2021), the decrease in SDW and RDW stopped cadmium concentrations in the *P. indica*-inoculated alfalfa plants. Similarly, according to Hassan et al. (2019), Ni reduced SL and RL and even caused a remarkable decline in other morphological traits. Rajput et al. (2022) further reported that the seed germination of plants had been diminished by Ni stress, while GR had not been reduced under different levels of Ni stress due to the application of *P. indica*, but it increased from 5 to 21% in the present study. The *P. indica*-treatment further boosted the growth of the tomato plants under Ni stress, indicating the effective role of *P. indica* in protecting the plants against the effects of Ni. According to Adya et al. (2013), *P. indica* had elevated nutrient uptake, allowing the plants to survive under abiotic stress and admit tolerance to heavy metal toxicity, along with other useful effects, such as growth improvement and favorable yield performance. Similar investigations have also reported the positive role of *P. indica* in inducing the tolerance of plants to copper (Sabra et al., 2018) and arsenic (Ghorbani et al., 2021) toxicity. Besides, Ghorbani et al. (2021) found that *P. indica* had enhanced plant growth with the inhibition of huge damage occurring to photosynthetic organs because arsenic could be immobilized in the roots and had not transferred to the plant shoots.

Treating *P. indica* correspondingly thwarted the harmful effects of Ni on H_2_O_2_ and decreased the adverse effects of Ni. The H_2_O_2_ magnitude also increased in the leaf tissue of *Solanum Lycopersicum* L. under Ni stress (Nazir et al., 2019), and it was expected that compounding the Ni concentrations could significantly elevate the quantities of H_2_O_2_, but treating the tomato plants with *P. indica* prevented its production in cells. The H_2_O_2_ production under heavy metal stress could also do considerable damage to cells and even lead to death (Das and Roychoudhury, 2014), while the antioxidant enzymes, like CAT and GPx, could scavenge H_2_O_2_, cause cell death, and provide defense against disbalances in the redox position of the stressed plants (García-Caparrós et al., 2021).

Furthermore, the study findings revealed that CAT and GPx significantly restricted H_2_O_2_ production under Ni-induced stress, but positive associations existed. The application of *P. indica* on tomato plants seemed to stimulate their defense mechanisms, like the high production of antioxidant enzymes. Reduction in CAT activity under Ni stress was also expected because the Ni excess could reduce some micronutrients, like iron or zinc, contributing to the structure of metalloenzymes, such as CAT (Altaf et al., 2021). The decrease in CAT activity recorded in this study was thus in agreement with the reports by Nazir et al. (2019) on tomatoes and Ghorbani et al. (2021) on rice.

Moreover, Chl a, b, as well as Carrot in the Ni-stressed plants treated with *P. indica*, were analyzed, suggesting that the amount of these pigments was significantly higher in the treated plants as compared with the non-treated ones grown under the same conditions. It seemed that *P. indica* could maintain the process of photosynthesis under Ni stress by inducing the defense system of the antioxidant enzymes, which could subside the Ni-induced oxidative stress. Such similar results have already been reported for tomatoes (*Solanum Lycopersicum* L.) (Ghorbani et al., 2018) and *Dimocarpus longan* seedlings (Cheng et al., 2022). There are also some antioxidant enzymes in plants, and it is expected that the *P. indica* inoculation can stimulate some of them, though the CAT activity decreased. However, only the enzyme activity was considered here, so the amount of enzyme might have amplified in the periods after inoculation through the rise in the expression of the related genes.

Given that antioxidant enzymes alone may not be very effective in cleaning ROS, the possibility of augmenting other enzymes that could scavenge ROS besides CAT is very high. Antioxidant enzymes and other non-enzymatic antioxidants also work together to reduce oxidative stress. Therefore, downgrading or escalating the activity of an antioxidant enzyme cannot be the reason for the success or failure of a plant defense process under oxidative stress. Therefore, *P. indica* aided the tomato plants by protecting them against Ni stress, but further investigations are required to grasp the mechanism of how *P. indica* is useful for plants. Although most studies have not simulated field conditions, the report by Khalid et al. (2019) supported the idea of using *P. indica* under field conditions for inducing tolerance to abiotic stress. Comparatively, the higher growth characteristics in the *P. indica*-treated plants could be attributed to their capacity to maintain higher Chl contents because the treated plants could delay photoinhibition under stress conditions and prevent the Ni interference in the photosynthetic electron transport chain and the light-harvesting complex II (Dubey, 2018). Moreover, the lower Chl a, b ratio recorded in the greenhouse and aeroponic experiments here under 300 mg L^−1^ of Ni stress and the greenhouse experiment under 900 mg L^−1^ of Ni stress demonstrated less damage to Chl b, indicating the better protection of the photosystem II (PSII) under the Ni-induced stress conditions (Janečková et al., 2019; Muhammed et al., 2022).

One other way for plants to protect themselves against Ni stress is the synthesis of osmolytes, such as Pro, which stabilizes proteins and membranes (Khan et al., 2020). In this study, the Pro quantities increased as Ni stress rose from 0 to 900 mg L^−1^, consistent with previous reports (Nazir et al., 2019). The increase in Pro may be further associated with reduced oxidative stress, resulting in the prevention of protein hydrolysis. The *P. indica* application augments this natural response under different Ni stress conditions. The changes in the membrane lipid composition were also found in this investigation since MDA augmented when the tomato plants were exposed to different Ni concentrations, as reported earlier on pigeon peas and cucumbers (Khoshgoftarmanesh et al., 2014). The lowest magnitudes of the MDA accumulation recorded in *P. indica*-treated plants also verified that this fungus could significantly contribute to mitigating the oxidative damage caused by Ni by enhancing the antioxidant capacity and reducing Pro and MDA in leaf cells.

The PCA of all experiments also indicated that the PC1 axis divided the traits into main groups, i.e., Pro, MDA, Flav, Phen, H_2_O_2_, CAT, and GPx in one group as an indicator of the Ni stress condition and the other traits in another group as a sign of normal or non-stress conditions. These results were in good agreement with the reports of Kirova et al. (2021), investigating wheat genotypes with similar templates of drought stress response associated with Pro, H_2_O_2_, Phen, Flav, CAT, and GPx. Although some traits in each group indicated weak or near-zero correlations, all had the same characteristics, such as common behavior under stress or non-stress conditions. Similarly, Plazas et al. (2019) reported that Pro, MDA, Flav, CAT, and GPx were involved in the stress response. The higher magnitudes of CA and GPx had also been recorded in the leaves of tolerant cultivars under drought stress, and these H_2_O_2_-scavenging enzymes were important in stress conditions (Kirova et al., 2021). The study results provide relevant information about the responses of the tomato plants to Ni stress, as well as the significant, positive effects of *P. indica* treatment on the removal of the harmful impacts of stress under different growing systems. It was thus suggested to evaluate commercial tomato cultivars inoculated with *P. indica* under Ni or other heavy metal stress conditions in farming fields to demonstrate the good effects of *P. indica* on meeting the goals of sustainable agriculture.

## 5. Conclusion

**Figure 8:**
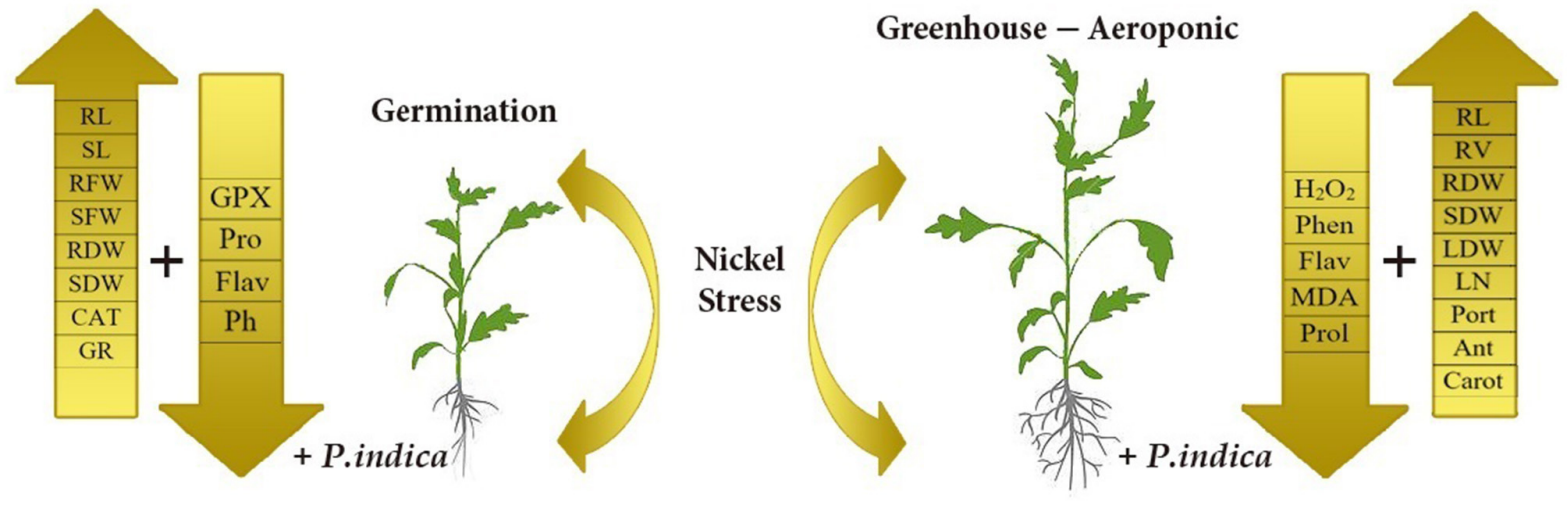
Schematic representation depicts morphological and physiological differences between *P. indica* colonized and uncolonized tomato plants. The overall health and productivity are enhanced *P. indica* infested tomato plants as compared to the non-infested ones under nickel stress.

This study revealed that *P. indica* treatment significantly and positively enhanced most measured traits, especially photosynthetic factors, and improved the growth performance of tomato plants under Ni toxicity stress. The application of *P. indica* also decreased the content of MDA and H_2_O_2_ and, as a result, increased the tolerance of the tomato plants under Ni exposure. The PCA also separated Pro, MDA, Flav, Phen, H_2_O_2_, CAT, and GPx as the stress-related traits from the others under Ni-induced stress and normal conditions (Figure 8). Considering the positive effects of *P. indica* on tomato tolerance to Ni toxicity, its application can thus be recommended as an eco-friendly tool and a plant biostimulant in Ni-contaminated regions.

## Data availability statement

The datasets presented in this article are not readily available because there is no restrictions. Requests to access the datasets should be directed to zahra_movahedi_312@yahoo.com.

## Author contributions

ZM and MG: conceptualization, methodology, and analysis. NM, ZM, and MG: validation and investigation. ZM: writing the original draft, reviewing, and editing the manuscript. All authors have read and agreed to the published version of the manuscript.
